# Efficient Motherhood

**Published:** 1913-04

**Authors:** Margaret Holliday

**Affiliations:** Austin, Texas


### Efficient Motherhood,

This is a subject upon which volumes could be written and then not give expression to all that might be said, but the essential elements can be dealt with more briefly.

It seems to me that the elements that go to make up efficiency for the vocation of parentage are three. There should be physical, mental and moral efficiency. The efficiency of motherhood and fatherhood is a question that concerns not only the welfare of the individual, but also the welfare of our race. An individual's parentage is the ladder upon which one climbs to higher things.

When we state that a woman, in order to be an efficient mother, should be physically fit, we do not mean to imply that only women of the amazon type should be selected or elected for such an office. The physically fit woman is the one with a good constitution, strong vitality, which necessitates the absence of weakness or disease transmittible by heredity. This of course would cause an investigation of the family history as regards physical condition, especially the presence of transmittible disease in her ancestry—tuberculosis, syphilis. After the fitness of the woman has been determined her search does not cease for the physical fitness of the man who is to be the father of her children must be investigated, and here both the life history of the individual and that of his forebears must be scrutinized.

The mental element in this efficiency for motherhood does not cease on the absence of mental diseases—insanity, inebriety, idiocy, weak-mindedness—this is very essential and is so essential that one cannot understand how a State does not protect her citizenship by laws ...................... Granted that there is no mental disease or weakness, but the mental efficiency is not established. It must be remembered that it is not enough that young offsprings have untainted minds in sound bodies but that they have to be trained and developed in order to be useful members of society. The woman who is to be an efficient mother must have this sound mind trained before she can be responsible for the development of another mind. This does not mean that only college-bred women should be allowed the privileges and responsibility of motherhood. We would be glad were it possible that every mother should be an educated woman, but "going through college" does not mean always that the mind has been trained. A mind can be trained by much thinking, self-analysis, right judging and judicial acting without ever having been even on the outside of a college. A woman capable of carrying a train of thought, who thinks clearly, does not become lost in the mire of emotions, who decides definitely and acts as the result of decision is fit to be a mother regardless of the presence or absence of college degrees. If she can come up to this mental test she is fitted to be the guiding mind in the development of her children and is that much more an efficient mother than the woman who merely feeds and clothes the young animals. By this I do not mean to disparage the importance of feeding and clothing, but the feeding and clothing by an efficient mother has more than the covering of the body or the satisfying of the pangs of hunger. It takes a woman of brains, education and much thought to feed and clothe children properly and efficiently.

The third essential or moral efficiency is so clearly interwoven with the mental characteristics that it is often difficult to separate them. In order to be an efficient parent a woman (or man) must have character. By this we mean that the individual must have some opinions in regard of thinking, acting and doing and must have the will power to live up to them. Opinions that are figure heads will never affect any one's character be it the parent herself or the offspring. If a woman has no opinions that are principles because they are adhered how under heaven is she to rear children possessing character—the ability to have principles and the will power to live up to them?

This is no Utopian dream. In order to have any results, a standard must be established and then striven for and the nearer the attributes of this standard is reached, the nearer will we come to the time when we will have efficient mothers and the more efficient the mother the more is she going to demand the same efficiency—physical, mental and moral—in the father.

MARGARET HOLLIDAY, M. D.

Austin, Texas, April 1, 1913.

